# Investigation of soil microbiome under the influence of different mulching treatments in northern highbush blueberry

**DOI:** 10.1186/s13568-021-01294-6

**Published:** 2021-09-28

**Authors:** Sang In Lee, Jungmin Choi, Hyunhee Hong, Jun Haeng Nam, Bernadine Strik, Amanda Davis, Yongsun Cho, Sang Do Ha, Si Hong Park

**Affiliations:** 1 Department of Food Science and Technology, Oregon State University, 3051 SW Campus Way, Corvallis, OR 97331 USA; 2 Department of Horticulture, Oregon State University, Corvallis, OR USA; 3 Department of Food Science and Technology, Chung-Ang University, Anseong, Gyeonggi-do Republic of Korea

**Keywords:** Soil microbiome, Mulch type, PGPR, Bacterial function

## Abstract

Microbial communities on soil are fundamental for the long-term sustainability of agriculture ecosystems. Microbiota in soil would impact the yield and quality of blueberries since microbial communities in soil can interact with the rhizosphere of plant. This study was conducted to determine how different mulching treatments induce changes in soil microbial composition, diversity, and functional properties. A total of 150 soil samples were collected from 5 different mulch treatments (sawdust, green weed mat, sawdust topped with green weed mat, black weed mat, and sawdust topped with black weed mat) at 3 different depths (bottom, middle, and top region of 20 cm soil depth) from 2 different months (June and July 2018). A total of 8,583,839 sequencing reads and 480 operational taxonomic units (OTUs) of bacteria were identified at genus level. Eight different plant growth promoting rhizobacteria (PGPR) were detected, and the relative abundances of *Bradyrhizobium*, *Bacillus*, and *Paenibacillus* were more than 0.1% among all soil samples. Sampling depth and month of soil samples impacted the amount of PGPR, while there were no significant differences based on mulch type. Functional properties of bacteria were identified through PICRUSt2, which found that there is no significant difference between mulch treatment, depth, and month. The results indicated that sampling month and depth of soil impacted the relative abundance of PGPR in soil samples, but there were no significant differences of functional properties and beneficial microbial communities based on mulch type.

## Key points

- Analysis of soil microbiome in five different soil mulch types
- Bacterial functions in soil were predicted
- Mulch treatment did not significantly affect on soil microbiome

## Introduction

Blueberries are the fruits obtained from two major blueberry species in the US market, the highbush (*Vaccinium corymbosum* L*.* and hybrids of *Vaccinium corymbosum* and *Vaccinium darrowi*) and lowbush (*V. angustifolium L.*) species (Wan et al. 2012). Highbush blueberries are cultivated in almost all of the North America, while lowbush blueberries are only produced commercially in eastern Canada and the northeastern US (Kang et al. 2015). Blueberries are a class of fruits having bioactivities which high in anthocyanins, phenolic acids, flavonoids, and flavan-3-oils. It has been reported that some bioactivities may manipulate antioxidant activity, antitumor, anti-inflammation and modulatory effects on a variety of cancer cells, acting on specific kinase-regulated pathways. Since anthocyanins in blueberries were identified as an antioxidant in human cells, which can provide beneficial effects on human health (Bornsek et al. 2012). Compared to other fruits and vegetables, a high antioxidant ability has been reported for lowbush blueberries (Conner et al. 2002; Kalt et al. 2000; Wang et al. 2017). With a continuous growing consumer’s interest in health-improving related foods, blueberry production is increasing worldwide (Caspersen et al. 2016). In addition, highbush blueberry was shown to inhibit carbohydrate hydrolyzing enzymes such as α-amylase and α-glucosidase, which can provide positive effects for type-2 diabetes (Johnson et al. 2011).

In recent years, the term ‘plant microbiome’ has received substantial attention since it influences both plant health and productivity (Lakshmanan et al. 2014). As it becomes rapid and easy how microbiome can influence ecosystems, there is a growing interest in microbiomes for shaping microbiota to alter ecosystems of interest (Loon et al. 2017). The plant microbiota is no longer considered as a single system, but rather dynamic entities comprising both plants and the soil microbial communities with complex interactions and functions (Vandenkoornhuyse et al. 2015). Plant-related microbiome research is important for improving human health and enhancing agricultural productivity.

Plant growth promoting rhizobacteria (PGPR) beneficially canonize the surface of plant roots and are known to influence plant growth by various direct or indirect mechanisms (Moncada et al. 2021). When it comes to plants, the rhizosphere and plant roots are continuously influenced by each other through the rhizodeposition which is an important interface process of the exchange and balance of the carbon among plant, soil, and microorganisms (Jones et al. 2004; Moe 2013). For these reasons, the Earth Microbiome Project (EMP) was launched in August 2010, to process the microbial diversity and functional potential from approximately 200,000 environment samples to understand the microbial properties of soil samples (Gilbert et al. 2014).

Weed management in blueberry farms is critical for the economic side since the presence of weeds contributes to decreasing the yield of the crop (Krewer et al. 2008). Mulching is widely used in agricultural areas, mainly because of their effectiveness for weed control (Julian et al. 2012; Strik et al. 2017). Application of mulch to the inrow area in blueberry farms improves production by improving weed control, holding soil moisture, and enhancing plant growth (Burkhard et al. 2009; Strik et al. 2020). Mulching with sawdust or a combination of sawdust and compost provided plant-available cations and increased soil organic matter compared to a black, woven polypropylene ground cover (weed mat) placed over bare soil (Larco et al. 2011; Strik et al. 2019). Blueberry farms in the northwestern US commonly used a mulch of douglas fir sawdust [*Pseudotsuga menziesii* (Mirb.) Franco], but in-row mulching with a black weed mat is now most common (Strik et al. 2021). Mulches may also affect root growth differently by altering the moisture and temperature in the soil (Strik et al. 2020). A sawdust topped with weed mat was more economical for weed control than weed mat over bare soil due to a positive effect on productivity (Strik et al. 2021). Currently, several colors of woven polypropylene weed mat are available for weed control, including black, white, and green (Strik et al. 2020).

The objective of this study was to evaluate the impact of different mulches, including sawdust, black or green weed mat, and sawdust covered with black or green weed mat on soil microbiome composition, functional analysis, and PGPR changes, which can potentially influence on blueberry plants.

## Materials and methods

### Experimental site

The experimental research site, establishment and management practices were described in a previous study (Strik et al. 2020). Briefly, the 0.14-ha area was located at the North Willamette Research and Extension Center (NWREC) of Oregon State University (OSU) in Aurora, OR (lat.45°16′47″ N, long.122°45′23″ W). The soil was composed of a Willamette silt loam (a mixed fine-silty, superactive mesic Pachic Ultic Argixeroll). A 5–8 cm deep layer of douglas fir sawdust was applied to the in-row area and mixed with the soil to a depth of around 20 cm. No fertilizer amendments were applied. Raised beds were formed using a bed shaper (1.2 m and 0.6 m wide at the base and top, respectively). Eighteen-month-old ‘Duke’ blueberry plants were planted on October 4th, 2016 at a standard spacing 0.9 m between plants and 3 m between rows. Mulch treatments were applied on top of the raised beds and included an 8-cm-deep layer of douglas fir sawdust, black weed mat (Baycor, Ten Cate Nicolon, Pendergrass, GA), green weed mat (Guerner & Irmãos, Perosinho, Portugal), and black or green weed mat over a 5-cm deep layer of sawdust. In each case, weed mat was installed in a “zippered” system with two 1-m-wide panels overlapping at the middle of the beds. Holes were cut in the weed mat around the crown of the plants. The black and green weed mat had a density of 108 and 130 g/m^−2^ and water infiltration rate of 407 and 554 L/m^−2^/min^−1^, respectively. Plots of each treatment were arranged in a completely randomized block design with five replicates. Each plot included a row of nine plants and was separated from adjacent plots in the row by 3 m.

### Soil sample collection

**Fig. 1:**
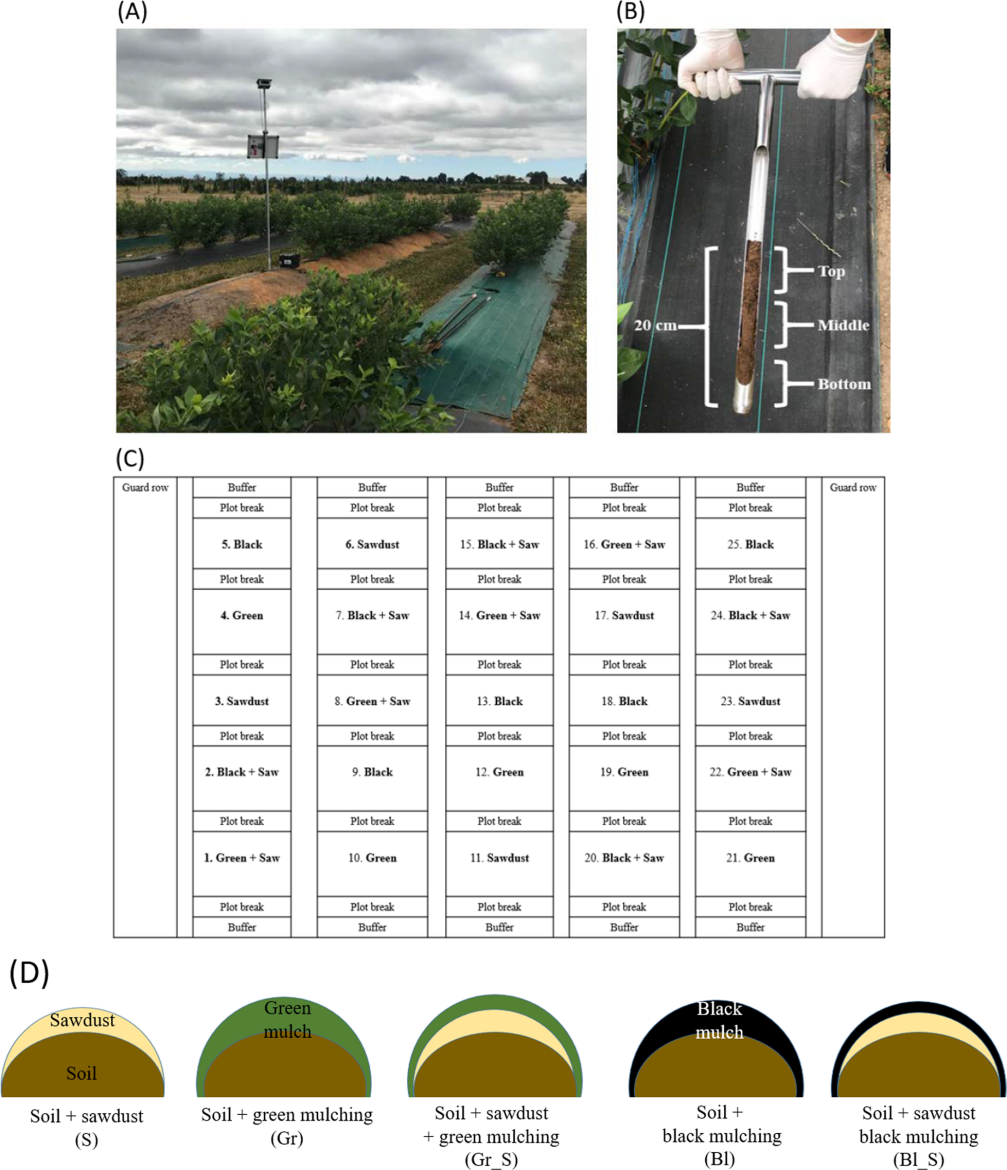
Sampling strategy for soil at the blueberry trial site. **A** blueberry trial site located at the NWREC in Aurora, OR, **B** 0–20 cm soil layers using a soil sampler probe which divide three different even depth, **C** random split-plot design for five different mulch treatments, and **D** mulch treatment design. *S* sawdust, *Gr* green weed mat, *Gr-S* sawdust topped with green weed mat, *Bl* black weed mat, and *Bl-S* sawdust topped with black weed mat

The soil samples at blueberry research site (Fig. 1A) were collected from 0 to 20 cm layers using a soil sampler probe, and soil samples were divided into three different depths (bottom, middle, and top) (Fig. 1B). To investigate the effect of mulch treatment on the soil microbial communities, the five treatments were established using a split-plot design with randomly allocated 5 replicated plots per treatment (Fig. 1C). The mulch types are designated as sawdust (S), green weed mat (Gr), sawdust topped with green weed mat (Gr-S), black weed mat (Bl), and sawdust topped with black weed mat (Bl-S) (Fig. 1D). A total of 150 soil samples [5 treatments × 5 replicated plots × 3 depths (bottom, middle, and top) × 2 times (June and July)] were collected and stored in 50 ml sterile tubes at -20°C until further experiments.

### DNA extraction

Bacterial and fungal DNA were extracted from 0.5 g of soil using a DNeasy PowerSoil DNA Kit (Qiagen, Hilden, Germany) according to the manufacturer’s instruction. The concentration of isolated DNA was measured via a Qubit 4 Fluorometer (Thermo Fisher Scientific, Waltham, MA, USA), and subsequently diluted with DNase-RNase free water to achieve a final concentration of 10 ng/µl.

### Library preparation

The microbial sequencing library was prepared targeting the V4 region of 16S rRNA gene for bacteria based on previous report (Kozich et al. 2013). In brief, extracted DNA from each sample was amplified using a high-fidelity AccuPrimeTM *Pfx* SuperMix (Thermo Fisher Scientific) and PCR products were confirmed through 1% agarose gel electrophoresis. Amplified PCR amplicons were normalized using a SequalPrep™ Normalization Plate Kit (Thermo Fisher Scientific) according to the manufacturer’s recommendation to standardize an equal amount of DNA (1–2 ng/µl). Following normalization, 5 ul of each normalized aliquot were combined into a pooled sample to construct a DNA sequencing library, and quantify a concentration via a KAPA Library Quantification Kit (Kapa Biosystems, Woburn, MA, USA). Finally, the library was diluted to the appropriate concentration prior to sequencing via MiSeq (Illumina, San Diego, CA, USA).

### Microbiome sequencing via an Illumina MiSeq platform

20 nM of pooled 16S rRNA gene library and 20 nM of PhiX control v3 (Illumina) were mixed with 0.2 N of fresh NaOH and HT1 buffer (Illumina) to produce the final concentration at 7.8 pM. The resulting library was mixed with the PhiX control v3 (10%, v/v, Illumina) and 600 µl loaded on a MiSeq® v2 Reagent cartridge (500 cycle, Illumina) for sequencing.

### Data analyses

Both demultiplexed R1 and R2 microbial raw sequences were acquired directly from the Illumina BaseSpace website (https://basespace.illumina.com/dashboard) and sequences were analyzed via a Quantitative Insights into Microbial Ecology 2 (QIIME 2, version 2020.11) open source pipeline (Bolyen et al. 2019). Demultiplexed sequences were joined and denoised for quality control via DADA2 scripts, available in QIIME 2, to generate a feature table for further analysis including measurement of community richness via Pielou’s evenness index. The processed sequencing data were compared to reference bacterial data from the GreenGenes (V.13.8) (http://greengenes.lbl.gov). For further statistical analysis and visual exploration, an operational taxonomic unit (OTU) table with taxa in plain format and metadata file was uploaded to the MicrobiomeAnalyst tool available at http://www.microbiomeanalyst.ca (Dhariwal et al. 2017). Additionally, the functional content of microbiome data from all soil samples was predicted using a Phylogenetic Investigation of Communities by Reconstruction of Unobserved States 2 (PICRUSt 2) (Langille et al. 2013). Alpha diversity, prevalence of three major PGPR and functional potential were compared statistically via analysis of variance (ANOVA) test.

## Results

### Taxonomic analysis of soil microbiome

After quality control, a total of 8,583,839 V4 region of 16S rRNA gene amplicon sequences were generated from 150 samples, including five mulching treatments (S, Gr, Gr-S, Bl, and Bl-S) with different months (June and July 2018) and depths (bottom, middle, and top) from each of five replicated plots. The mean value for the frequency of bacterial sequences per sample was found to be 57,226 reads after data was analyzed using a QIIME2. A total of 480 OTUs for bacteria were identified in the genus level.

**Fig. 2:**
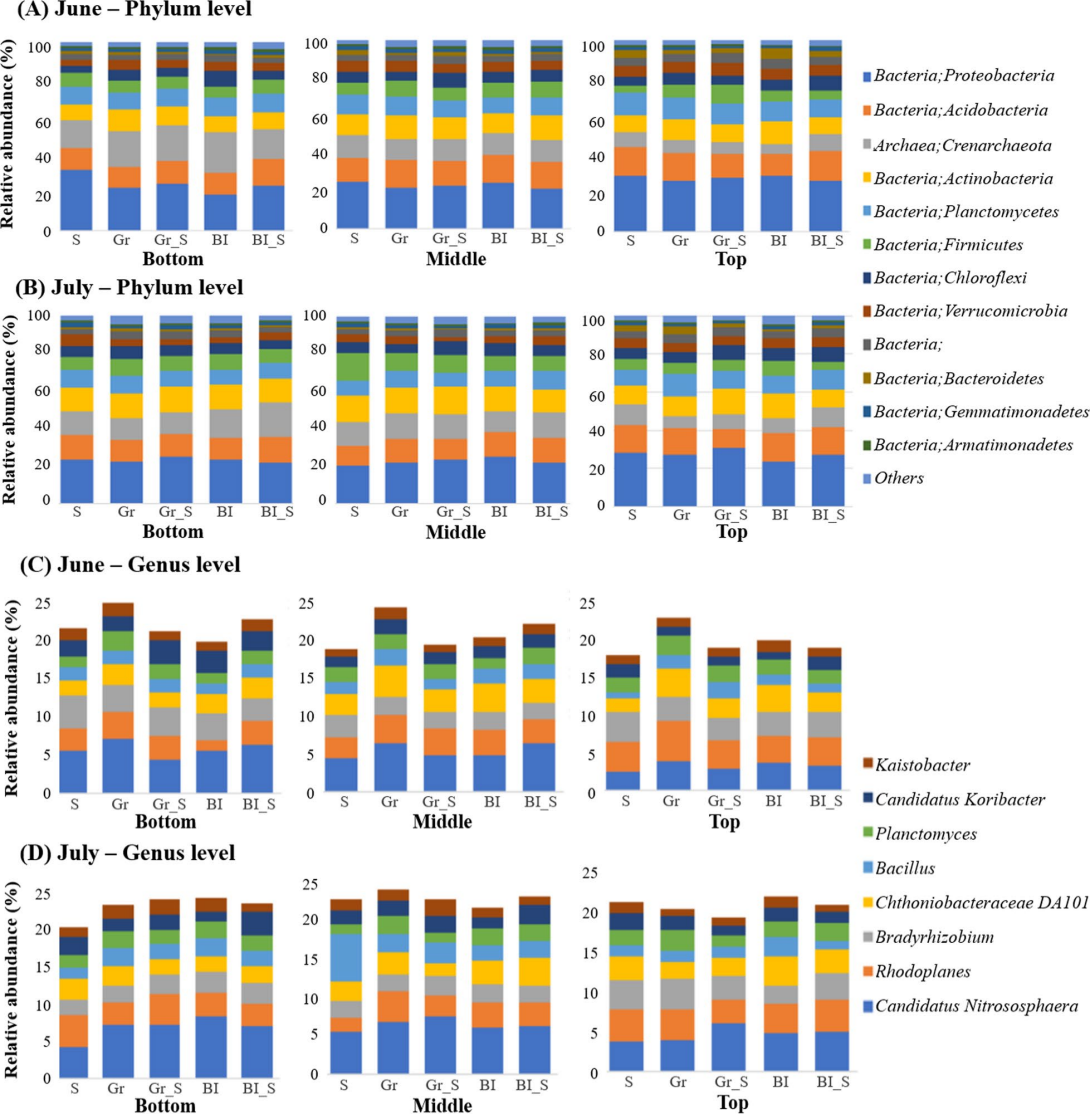
The most abundant taxa in phylum level (more than 1%) of bacteria in 5 different mulched soil; bacterial communities from **A** June and **B** July The most abundant taxa in genus level (more than 1%) of bacteria in 5 different mulched soil; bacterial communities from **C** June and **D** July. *S* sawdust, *Gr* green weed mat, *Gr-S* sawdust topped with green weed mat, *Bl* black weed mat, and *Bl-S* sawdust topped with black weed mat

Overall, list of the detected top 10 bacterial taxa from phylum level were shown in Fig. 2A and B. In brief, *Proteobacteria* (24.7%), *Acidobacteria* (13.1%), *Crenarchaeota* (11.9%), *Actinobacteria* (11.5%), *Planctomycetes* (9.5%), *Firmicutes* (7.3%), *Chloroflexi* (6.0%), *Verrucomicrobia* (4.7%), *Bacteroidetes* (1.8%), and *Gemmatimonadetes* (1.7%) were the 10 most abundant bacterial taxa at the phylum level. The most abundant bacterial genera with at least 1% of relative abundance were: *Candidatus Nitrososphaera* (5.4%), *Rhodoplanes* (3.4%), *Bradyrhizobium* (2.8%), *Chthoniobacteraceae DA101* (2.7%), and *Planctomyces* (1.9%) (Fig. 2C and D). Among all the bacteria at genus level, *Bradyrhizobium* was the most abundant plant growth promoting rhizobacteria (PGPR).

### PGPR prevalence in soil

PGPR are soil bacteria inhabiting the root surface and directly or indirectly involved in promoting growth and development of plants (Ahemad and Kibret 2014). Based on the microbiome results, 8 different PGPR (*Bradyrhizobium*, *Bacillus*, *Paenibacillus*, *Mesorhizobium*, *Rhizobium*, *Azospirillum*, *Acinetobacter*, and *Pseudomonas*) were detected in soil samples. Among these 8 PGPR, the relative abundance of *Bradyrhizobium* and *Bacillus* were exhibited to be more than 1% for all samples (Table 1). According to the relative abundance of the 3 major PGPR, there was no significant effect of mulch treatment while the relative abundance of bacteria was dependent on sampling month and the depth of soil samples (*P* > 0.05). Especially *Bradyrhizobium* decreased in July from June in all treatments, but *Bacillus* and *Paenibacillus* showed an increasing trend (Table 1).

**Table 1:** Relative abundance (%) of PGPR identified in soil samples higher than 0.1% of all the soil samples

Category	Relative abundance (%)									
	June					July				
	S	Gr	Gr_S	Bl	Bl_S	S	Gr	Gr_S	Bl	Bl_S
PGPR										
* Bradyrhizobium*	3.67 ± 1.53	2.89 ± 0.79	2.87 ± 0.98	2.90 ± 0.92	2.57 ± 0.84	2.69 ± 1.18	2.76 ± 1.31	2.66 ± 0.81	2.53 ± 0.67	2.77 ± 0.87
* Bacillus*	1.43 ± 0.68	1.84 ± 0.66	1.74 ± 1.51	1.50 ± 0.75	1.66 ± 0.86	3.00 ± 4.90	2.07 ± 1.13	2.03 ± 0.86	2.28 ± 0.71	1.77 ± 0.71
* Paenibacillus*	0.62 ± 0.44	0.40 ± 0.40	0.46 ± 0.40	0.57 ± 0.42	0.53 ± 0.43	0.75 ± 0.59	0.49 ± 0.48	0.62 ± 0.53	0.77 ± 0.43	0.56 ± 0.39

No significant difference between groups (*P* > 0.05)

*S* sawdust, *Gr* green weed mat, *Gr-S* sawdust topped with green weed mat, *Bl* black weed mat, and *Bl-S* sawdust topped with black weed mat

### Alpha diversity

**Fig. 3:**
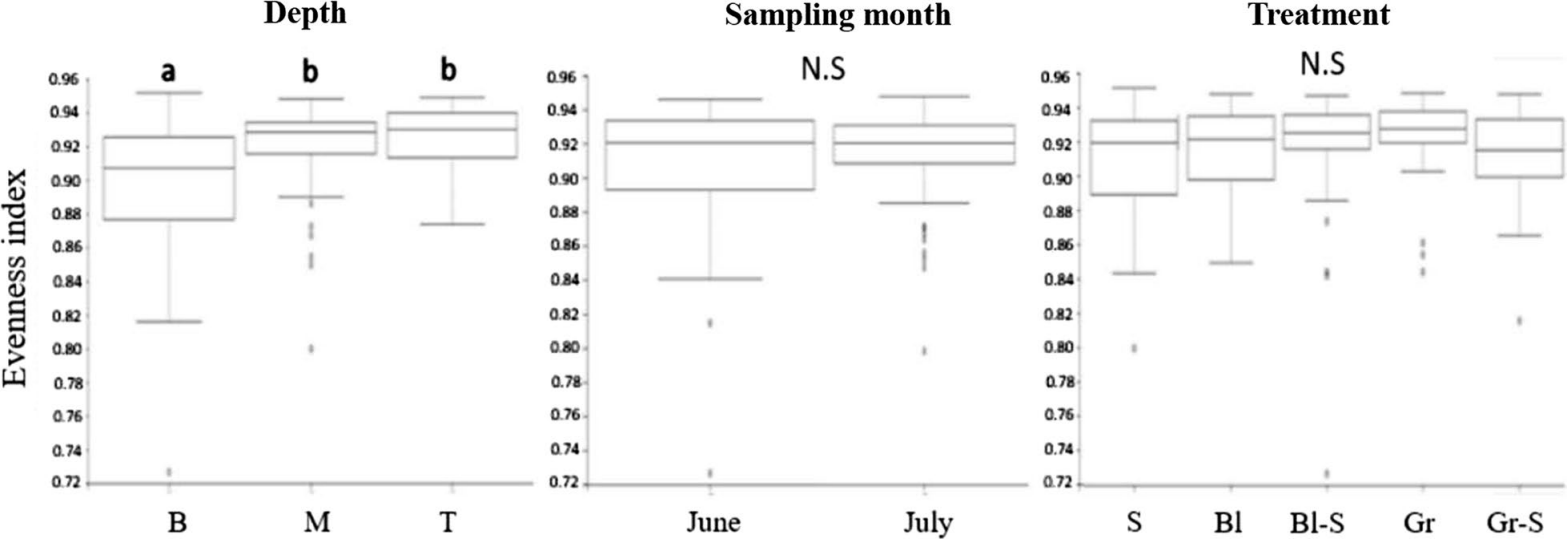
Alpha diversity (evenness) of bacteria between different sampling depth, month, and mulch type in soil samples. *B* bottom, *M* middle, *T* top, *S* sawdust, *Gr* green weed mat, *Gr-S* sawdust topped with green weed mat, *Bl* black weed mat, and *Bl-S* sawdust topped with black weed mat

Alpha diversity of the microbial communities was analyzed using the evenness which derived from the analysis generated by QIIME 2 (https://qiime2.org/). The evenness indicated how microbial communities of each species are close to one another in an environment. When it comes to analyzing alpha diversity, the evenness of sampling month and mulch treatment showed no significant differences (*P* > 0.05) (Fig. 3). However, according to depth, the bottom region exhibited a significant low evenness compared to the middle or top region of soil (*P* < 0.05) (Fig. 3).

### Functional analysis

In order to compare functional potentials based on different mulching treatments, depths, and months, functional contents were predicted from 16S rRNA marker gene using PICRUSt2. Several predicted pathways were significantly enriched in the microbiome; carbohydrate metabolism, amino acid metabolism, energy metabolism, metabolism of cofactors and vitamin, nucleotide metabolism, lipid metabolism, xenobiotics biodegradation metabolism, metabolism of other amino acids, metabolism of terpenoids and polyketides, glycan biosynthesis and metabolism, and biosynthesis of other secondary metabolites (Table 2). According to the functionality of the soil microbiota, carbohydrate metabolism and amino acid metabolism were the most abundant more than 19% and biosynthesis of other secondary metabolites were the lowest abundant (2.2%) function. Other functional potentials of soil microbiome in June and July showed the similar ranges as follows: energy metabolism (14%), metabolism of cofactors and vitamins (12.7–12.8%), nucleotide metabolism (8.1%), lipid metabolism (6.4%), xenobiotics biodegradation and metabolism (5.1–5.3%), metabolism of other amino acids (4.9–5%), metabolism of terpenoids and polyketides, and glycan biosynthesis and metabolism (3.2–3.5%). Overall, functional potentials of each soil microbiome in June and July showed no significant differences (*P* > 0.05).

**Table 2:** Relative abundance of functional bacterial genes in soil samples with different mulch types

Functional potentials	Relative abundance (%)									
	June					July				
	S	Gr	Gr_S	Bl	Bl_S	S	Gr	Gr_S	Bl	Bl_S
Carbohydrate metabolism	19.69 ± 0.16	19.78 ± 0.25	19.77 ± 0.22	19.79 ± 0.26	19.81 ± 0.21	19.74 ± 0.15	19.92 ± 0.14	19.85 ± 0.18	19.85 ± 0.20	19.80 ± 0.17
Amino acid metabolism	19.53 ± 0.17	19.58 ± 0.19	19.58 ± 0.17	19.60 ± 0.19	19.59 ± 0.14	19.67 ± 0.16	19.60 ± 0.15	19.65 ± 0.16	19.65 ± 0.16	19.61 ± 0.18
Energy metabolism	14.09 ± 0.18	14.10 ± 0.20	14.15 ± 0.27	14.12 ± 0.35	14.13 ± 0.20	14.03 ± 0.22	13.98 ± 0.20	14.00 ± 0.22	14.03 ± 0.19	14.16 ± 0.17
Metabolism of cofactors and vitamins	12.79 ± 0.23	12.80 ± 0.17	12.78 ± 0.31	12.83 ± 0.38	12.80 ± 0.18	12.77 ± 0.14	12.68 ± 0.22	12.69 ± 0.19	12.70 ± 0.18	12.78 ± 0.23
Nucleotide metabolism	8.06 ± 0.27	8.12 ± 0.23	8.12 ± 0.23	8.19 ± 0.32	8.15 ± 0.13	8.15 ± 0.16	8.07 ± 0.23	8.05 ± 0.19	8.12 ± 0.16	8.14 ± 0.21
Lipid metabolism	6.45 ± 0.21	6.42 ± 0.24	6.42 ± 0.24	6.39 ± 0.32	6.38 ± 0.14	6.43 ± 0.15	6.43 ± 0.18	6.42 ± 0.16	6.42 ± 0.14	6.37 ± 0.19
Xenobiotics biodegradation and metabolism	5.31 ± 0.31	5.25 ± 0.29	5.25 ± 0.29	5.14 ± 0.32	5.12 ± 0.14	5.23 ± 0.21	5.30 ± 0.26	5.35 ± 0.20	5.26 ± 0.19	5.23 ± 0.24
Metabolism of other amino acids	4.99 ± 0.15	4.95 ± 0.14	4.95 ± 0.14	4.91 ± 0.17	4.92 ± 0.07	4.95 ± 0.11	4.95 ± 0.13	4.99 ± 0.10	4.94 ± 0.11	4.91 ± 0.10
Metabolism of terpenoids and polyketides	3.54 ± 0.07	3.54 ± 0.07	3.54 ± 0.07	3.54 ± 0.10	3.54 ± 0.06	3.57 ± 0.06	3.54 ± 0.04	3.56 ± 0.06	3.57 ± 0.07	3.54 ± 0.06
Glycan biosynthesis and metabolism	3.37 ± 0.19	3.29 ± 0.17	3.29 ± 0.17	3.33 ± 0.20	3.38 ± 0.16	3.27 ± 0.17	3.31 ± 0.16	3.22 ± 0.17	3.26 ± 0.18	3.27 ± 0.14
Biosynthesis of other secondary metabolites	2.17 ± 0.05	2.16 ± 0.05	2.16 ± 0.05	2.17 ± 0.04	2.18 ± 0.04	2.17 ± 0.05	2.21 ± 0.04	2.20 ± 0.04	2.18 ± 0.04	2.18 ± 0.04

No significant difference between groups (*P* > 0.05)

*S* sawdust, *Gr* green weed mat, *Gr-S* sawdust topped with green weed mat, *Bl* black weed mat, and *Bl-S* sawdust topped with black weed mat

### Microbial communities of soil on phylum level

**Fig. 4:**
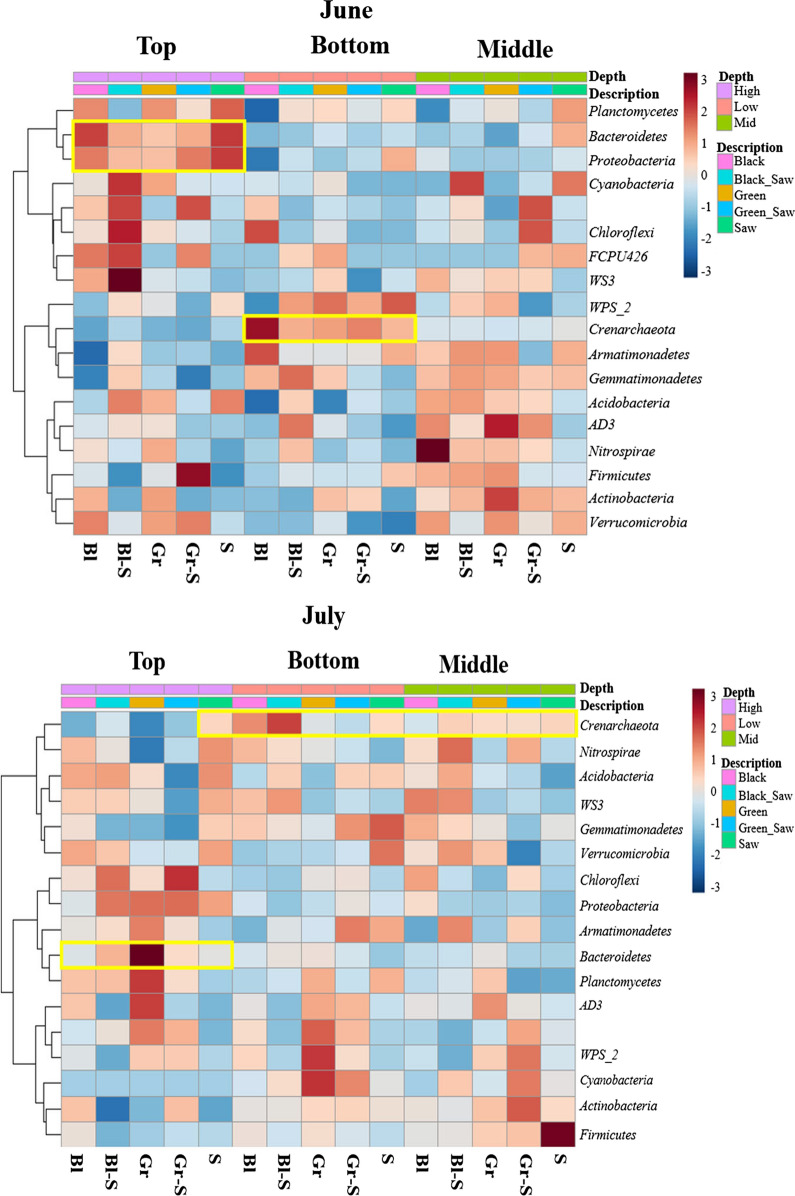
Heat map of microbial communities of soil based on phylum level. *S* sawdust, *Gr* green weed mat, *Gr-S* sawdust topped with green weed mat, *Bl* black weed mat, and *Bl-S* sawdust topped with black weed mat

Heat maps of the microbial community of five soil samples in June and July based on phylum level were determined (Fig. 4). The results of microbial communities in the soil samples exhibited a variety of values. For example, *Crenarchaeota* in the bottom region in June was remarkably higher than other regions while the *Crenarchaeota* in July was decreased but the middle region was increased. In the top area, *Proteobacteria* and *Bacteroidetes* in June were noticeably detected. In addition, *Bacteroidetes* in the top region was more identified than the bottom and the middle area in June and July.

### Dendrogram of soil microbiome

**Fig. 5:**
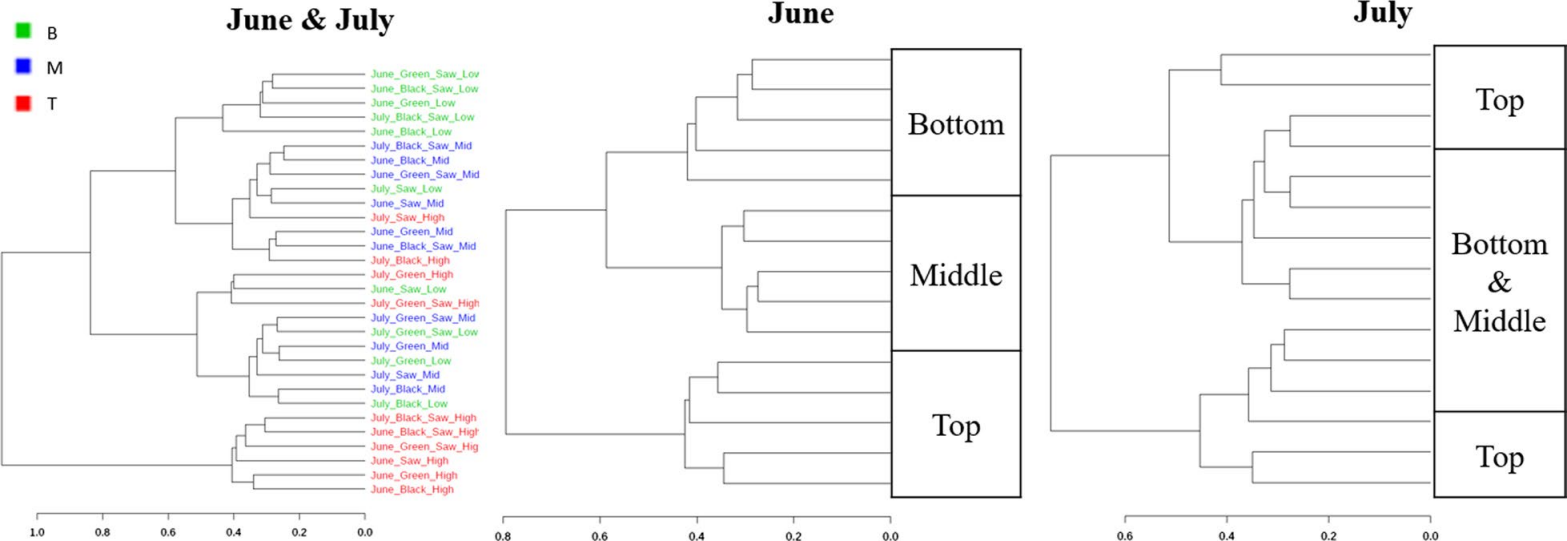
Dendrogram of soil microbiome in five different mulch types, depths, and months. *B* Bottom, *M* Middle, *T* Top

The dendrogram of soil microbiome in June and July were produced using Bray–Curtis Index (Fig. 5). Soil microbial community in each sampling depth was clustered in June while both middle and bottom regions were strongly clustered together in July.

## Discussion

The goal of our research was to analyze the bacterial populations from different mulching treatments, depths, and months. The soil that surrounds the plant roots is a primary source of the bacterial agents promoting plant growth. The analysis of bacterial communities from different mulching treatments and depths of soil samples provided a better understanding of how the microbial communities differ. Briefly, eight notable PGPR were identified that slightly differences in relative abundance depend on sampling depth and month. Although there was no statistical difference in PGPR composition and functional properties by treatments, sampling depth and month exhibited significant differences in *Bradyrhizobium* and *Bacillus*.

According to the previous study, the composition of bacterial communities in wild or cultivated blueberry soil samples, *Acidobacteria*, *Actinobacteria*, *Proteobacteria*, *Chloroflexi*, and *Bacteroidetes* were identified as the most relatively abundant bacteria (phylum level) corresponding to the findings in our study (Foulon et al. 2016; Yurgel et al. 2017). In the genus level, *Candidatus Nitrososphaera*, *Bradyrhizobium*, *Rhodoplanes*, *Chthoniobacteraceae DA101*, and *Planctomyces* were the most relatively abundant bacteria among all the soil samples, and among 5 most abundant bacteria, which is the at least one percent of relative abundance. The bacteria *Bradyrhizobium* is the PGPR which improves the growth of blueberry plants through nitrogen-fixing (Kaneko et al. 2002).

The PGPR, which are the bacteria living on or inside plant roots, exert highly beneficial effects on plant growth and development by direct or indirect mechanisms (Timmusk et al. 2017). When it comes to our microbiome analysis, *Bradyrhizobium*, *Bacillus*, *Paenibacillus*, *Mesorhizobium*, *Rhizobium*, *Azospirillum*, *Acinetobacter*, and *Pseudomonas* were identified as PGPR (Ahemad and Kibret 2014) and among 8 different PGPR, only *Bradyrhizobium*, *Bacillus*, and *Paenibacillus* showed at least 0.1% of relative abundance. While mulching treatment did not impact the PGPR composition, sampling months (June and July) and depths (bottom, middle, and top region) impacted the statistical differences between soil samples. For instance, the relative abundance of *Bradyrhizobium* in June samples was significantly decreased at the bottom region. Moreover, in June, the relative abundance of *Bradyrhizobium* from the middle region of Gr treated soil was significantly lower than the top and bottom region. Additionally, the relative abundance of *Bacillus* was also showed to be significantly increased in July. *Bradyrhizobium*, which is the most abundant PGPR among all the soil samples, can fix atmospheric nitrogen by converting it to nitrogenous compounds (Bogino et al. 2006). According to the Wani and Khan (2010), *Bacillus* is able to work as a nitrogen fixer and produce indoleacetic acid (IAA), which is the most common naturally occurring plant hormone to regulate the plant gene. Lastly, *Paenibacillus* species can promote crop growth directly via biological nitrogen fixation, phosphate solubilization, production of the phytohormone IAA, and release of siderophores that enable iron acquisition. They can also offer protection against insect herbivores and phytopathogens, including bacteria, fungi, nematodes, and viruses (Grady et al. 2016).

PICRUSt2 was used to predict the functional composition of the metagenomes from soils with different mulching treatments, depths, and months. The predicted abundance of these genes was compared between soil samples. Overall, carbohydrate metabolism and amino acid metabolism related functional genes were the most abundant in soil samples between bacterial communities from soil, and biosynthesis of other secondary metabolites and glycan biosynthesis and metabolism were the lowest functional genes from soil samples. In order to understand how mulching treatments, sampling depths and months impacted the functional gene abundance between microbial communities in soil samples, we compared the relative abundance of functional genes, but there were no significant differences observed between samples.

Wang et al. (2020) identified that mulching practices in apple trees can influence soil quality with microbial communities and four treatments (conventional tillage, intercrop ryegrass cover, inter-row cornstalk mulch, and black ground fabric mulch). The comprehensive results of our study were different from this previous study in that the previous study exhibited significant differences in bacterial richness and increased the OTU abundance. Different mulching treatments, however, may influence the results of these studies. Furthermore, the soil samples of this study were prepared by different depths and two months periods, whereas the previous study collected soil samples in each different soil in apple trees. Study by Chen et al. (2019) where alpha diversity of rhizosphere and non-rhizosphere were compared, non-rhizosphere soil exhibited more diverse, or less even, microbial diversity which suggest bottom region of soil may too far from the plant roots as rhizosphere that resulted lower evenness of diversity in the present study. Moreover, the results of the present study may be influenced by a variety of factors, such as climate, soil properties, and the period of experiments. Chou et al. (2018) reported that soil microbial composition under vine from 2014 to 2016. According to the previous study, climatic conditions play a significant role in microbial structure by demonstrating climate differences as the key factor explaining variance in soil and grape fungal assemblage. As this present study was conducted in June and July, which is the warm and dry season in Oregon, it can agree with the previous study. In the future, there is a need to obtain effective data through long-term soil microbiome analysis. In addition, it is necessary to confirm the effect of mulching by additionally performing a functional study of each microorganism.

## Data Availability

The 16S rRNA sequences are available at the BioProject of the National Center for Biotechnology Information (NCBI); PRJNA743071.
